# Protocol to isolate nuclei from *Chlamydomonas reinhardtii* for ATAC sequencing

**DOI:** 10.1016/j.xpro.2023.102764

**Published:** 2024-01-18

**Authors:** Indu Santhanagopalan, Antonia Netzl, Tanya Mathur, Alison Smith, Howard Griffiths, Andre Holzer

**Affiliations:** 1 Department of Plant Sciences, University of Cambridge, Downing Street, Cambridge CB2 3EA, UK; 2 Department of Zoology, University of Cambridge, Cambridge CB2 3EJ, UK; 3 Institute of Molecular Plant Sciences, University of Edinburgh, Edinburgh EH9 3BF, UK; 4 Center for Bioinformatics and Department of Computer Science, Saarland University, 66123 Saarbrücken, Germany

**Keywords:** Genetics, Genomics, Sequencing, Model Organisms, Plant sciences, Molecular Biology, Systems biology, Environmental sciences

## Abstract

The isolation of sufficient amounts of intact nuclei is essential to obtain high-resolution maps of chromatin accessibility via assay for transposase-accessible chromatin using sequencing (ATAC-seq). Here, we present a protocol for tag-free isolation of nuclei from both cell walled and cell wall-deficient strains of the green model alga *Chlamydomonas reinhardtii* at a suitable quality for ATAC-seq. We describe steps for nuclei isolation, quantification, and downstream ATAC-seq. This protocol is optimized to shorten the time of isolation and quantification of nuclei.

## Highlights

• Optimized isolation of nuclei from the green model alga *Chlamydomonas reinhardtii*
• Tag-free isolation from both cell-walled and cell wall-deficient algae strains
• Key steps for an effective and fast isolation and quantification procedure of nuclei
• Extracts at a quality suitable for ATAC-sequencing

## Abstract (editor highlights)

Publisher’s note: Undertaking any experimental protocol requires adherence to local institutional guidelines for laboratory safety and ethics.

## Graphical abstract

**Figure:**
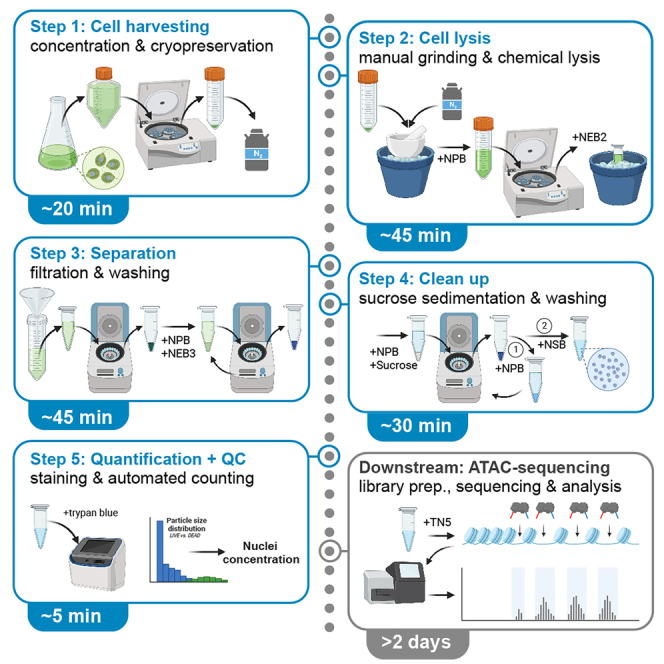

## Before you begin

ATAC-seq is a rapid and sensitive method to determine chromatin accessibility and thereby gain information on the global epigenetic landscape in the nucleus of eukaryotic cells.^1^ Originally established for clinical applications in human cells,^1^^,^^2^ ATAC-seq has gained popularity and has been successfully adapted to study the chromatin landscape of various eukaryotic model organisms, including *Drosophila melanogaster* (fruit fly),^3^ *Mus musculus* (mouse),^4^ *Aiptasia pallida* (sea anemone),^5^ *Arabidopsis thaliana* (thale cress),^6^^,^^7^ *Oryza sativa* (rice)^6^ and *Solanum lycopersicum* (tomato).^6^ The technique utilizes a hyperactive Tn5 transposase to cause DNA cleavage coupled with insertion of sequencing adapters into accessible regions of chromatin. The availability of good quality starting material of about 50,000 nuclei free from interfering organellar DNA is key to high quality ATAC-seq library preparation.^2^ Yet, the isolation of sufficient and clean nuclei poses difficulties, especially for organisms with cell walls, like plants and algae. For single-celled microalgae, the existence of strains with and without cell walls alongside their unique metabolic characteristics presents a significant challenge to perform downstream genomic experiments at chromatin level. While the lysis methods for nuclei isolation must be harsh enough to break the cell wall and organellar membranes, the method must not damage nuclear envelopes and denature nuclear proteins. With plants and algae containing plastids, contamination of extracts with plastid DNA can further lead to a decrease in ATAC-seq reads of nuclear origin. To circumvent these challenges in plants, the INTACT method (Isolation of Nuclei Tagged in specific Cell Types) which involves affinity purification of nuclei expressing a biotinylated nuclear envelope protein has been developed,^8^ and further optimized for use in plant ATAC-seq.^9^ INTACT requires to genetically modify cells of interest which is not available for many algae nor suitable for gaining insights on native strains. Bajic et al.^9^ also described a tag-free tissue lysis method with which sufficiently pure total nuclei can be isolated from plants, that provides the opportunity to follow a similar strategy for the isolation of nuclei from algae.

**Figure 1:**
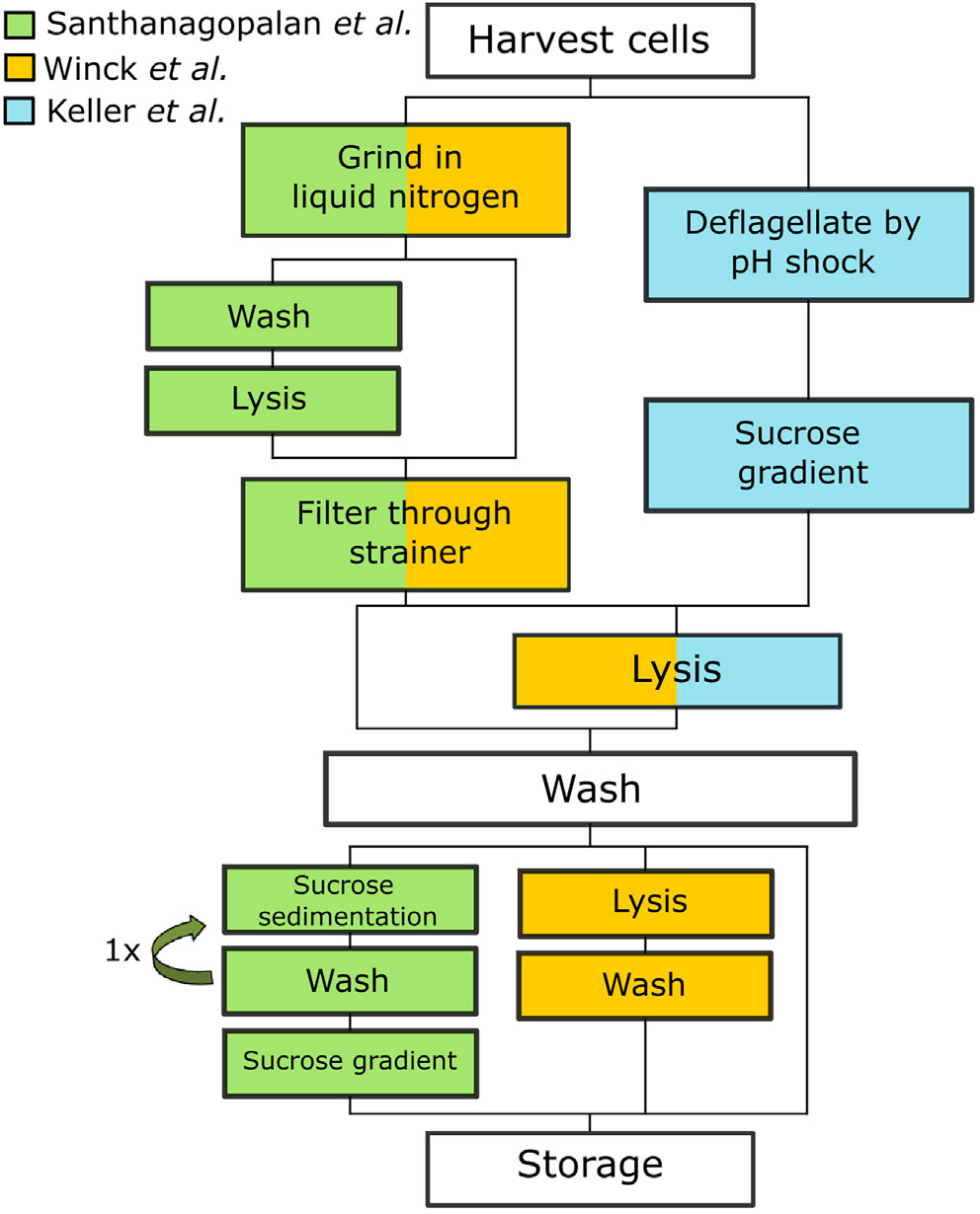
Schematic workflow comparison of the here described methodology to other algae nuclei isolation protocols Steps that are colored are specific to one protoc^24^ol, indicated by the underlying color (green: Santhanagopalan et al. (this article), yellow: Winck et al*.*,^23^ blue: Keller et al.). Steps that are common to all protocols are shown in white; steps that are shared between protocols are bi-colored. The required time per isolation, as determined by internal test runs, is 4 h for Winck et al. (4 samples), 5 h for Keller et al. (4 samples), and 2 h for Santhanagopalan et al*.* (2 samples).

The model green alga *Chlamydomonas reinhardtii* has been widely used to understand many aspects of cell and molecular biology^10^^,^^11^^,^^12^^,^^13^^,^^14^ including epigenetic mechanisms.^15^^,^^16^^,^^17^^,^^18^^,^^19^ Despite the availability of genomic information,^11^^,^^20^ extensive transcriptomics data^21^^,^^22^ and its widespread use in studying key biological processes, no ATAC-seq data has been published for this model organism yet. Although two methodologically different nuclei isolation protocols have been described for *C. reinhardtii* previously,^23^^,^^24^ those were not designed for the use of nuclei in next-generation sequencing (NGS) approaches and our attempts to replicate the methods did not produce the required quality of nuclei isolates. In this article, we present an optimized methodology for isolation of *C. reinhardtii* nuclei that extends on recent approaches to purify plant nuclei for ATAC-seq^9^ by integrating several key steps from other different protocols.^23^^,^^24^ The presented protocol has optimized filtration and resuspension steps following centrifugation, extended washing and sedimentation steps and an easy mode of quantification of nuclei (Figure 1). Our modifications allow for the preparation of clean and homogeneous nuclei from both cell walled and cell wall-deficient *C. reinhardtii* without the need for genetic tagging. Moreover, the processing time is much shorter at just 2 h, and the quantification method allows the transposase reaction to be set up within a few minutes following isolation of nuclei. We also tested the nuclei isolated from cell wall deficient *C. reinhardtii* by carrying out transposase reactions and library preparations and show that these nuclei are indeed ATAC-seq compatible.

The protocol below describes the specific steps to extract nuclei from *C. reinhardtii* for further use in downstream NGS approaches such as ATAC-sequencing. Important steps of the procedure are illustrated, using results obtained from processing descendants of the cell wall-deficient (CC-5390), and the cell walled (CC-124) strains of *C. reinhardtii* as an example.

Before you start with the nuclei isolation, make sure that all equipment and reagents listed in the key resources table are available. Ensure that the cell lines are ready for harvesting and prepare the stock solutions listed in materials and equipment. On the day of nuclei extraction, prepare all buffers and cool required equipment prior usage.

### Preparation I: Cultivate cell lines

**Timing: 3–4 days**

1. This protocol requires *C. reinhardtii* cells grown to log phase (in the range of 1–5·10^6^ cells/mL). In principle, any common *C. reinhardtii* strain or media can be used and various growth conditions can be applied. We have used several replicates of CC-124 and CC-5390 derived *C. reinhardtii* cells grown to log phase (1–1.5·10^6^ cells/mL) in TAP and HS media, respectively.

**CRITICAL:** Cultures should reach a density corresponding to log phase before harvesting.

### Preparation II: Prepare the stock solutions and equipment

**Timing: ∼6 h**

2. Follow the instructions provided in materials and equipment to prepare all 14 stock solutions.
3. Autoclave all stock solutions, except 80% ethanol, 100 mM spermidine, and 1 M DTT and store at room temperature. Sterilize the spermidine and DTT solutions using 0.22 μm syringe filter, and store at ‒20°C.
4. Autoclave TAP and HS media, pipette tips and the required number of spatulas and funnels.

***Note:*** 80% ethanol does not need to be sterilized.

**CRITICAL:** Make sure you use nuclease free water to prepare the stock solutions.

### Preparation III: Prepare the buffers

**Timing: ∼1.5 h**

5. On the day of nuclei extraction, prepare all buffers fresh following the recipes in the materials and equipment section. Sterilize them using 0.22 μm syringe filters.
6. Pre-chill 1 mL pipette tips, spatula, and funnels on ice inside their autoclaved bag/container before they are used.

## Key resources table

**Table:** 

REAGENT or RESOURCE	SOURCE	IDENTIFIER
**Chemicals, peptides, and recombinant proteins**		

Triton X-100	VWR	TRI002
β-Mercaptoethanol	VWR	MER004
Nuclease-free water	Thermo Fisher Scientific	10977035
Phenol:chloroform:isoamyl alcohol (25:24:1)(v/v)	Thermo Fisher Scientific	15593031
10X Phosphate-buffered saline (PBS), pH 7.4	Thermo Fisher Scientific	AM9624
MOPS	Sigma	M1254
EGTA	Sigma	324626
Spermidine	Sigma	S2626
Sodium hydroxide (NaOH)	Sigma	30620
Glycerol	Sigma	G5516
Tris	Sigma	10708976001
HEPES	Sigma	H3375
Complete protease inhibitor cocktail tablets	Roche	11697498001
2% Digitonin	Promega	G9441
Magnesium chloride hexahydrate (MgCl_2_⋅6H_2_O)	Merck	M2670
Dithiothreitol (DTT)	Melford Laboratories	D11000
Potassium chloride (KCl)	Honeywell	31248
2-Propanol	Honeywell	33539
Sucrose	Merck	S9378
Molecular biology grade Ethanol (200 proof)	Fisher BioReagents	BP2818100
Sodium chloride (NaCl)	Fisher	SODC002
Disodium salt of EDTA	Fisher	EDT003
Potassium hydroxide (KOH)	Fisher	POT014
Cetyltrimethylammonium bromide (CTAB)	Calbiochem	219374

**Experimental models: Organisms/strains**		

*Chlamydomonas reinhardtii* CC-5390	Chlamydomonas Resource Center, University of Minnesota (www.chlamydomonas.org)	CC-5390
*Chlamydomonas reinhardtii* CC-124		CC-124

**Software and algorithms**		

FastQC	Andrews^25^	https://www.bioinformatics.babraham.ac.uk/projects/fastqc/
Cutadapt	Martin^26^	https://anaconda.org/bioconda/cutadapt
Bowtie2	Langmead et al.^27^	https://anaconda.org/bioconda/bowtie2
SAMtools	Li et al*.*^28^	https://anaconda.org/bioconda/samtools
Integrated Genomics Viewer	Robinson et al.^29^	https://igv.org/

**Other**		

Diagenode ATAC-seq kit	Diagenode	C101080001
0.2 mL PCR tubes	Bio-Rad	TFI0201
384-well PCR plates	Bio-Rad	HSP3805
Plate sealing films	Bio-Rad	MSB1001
AMPure XP beads	Beckman Coulter, Inc.	A63881
50 mL tubes	BD Falcon	352070
20 mL and 50 mL syringes	BD	300613, 300866
Filter tips	Starlab	S1120-8810, S1122-1830, S1121-3810
Autoclaved miracloth (10 cm × 10 cm pieces, 20 μm)	Millipore	475855
0.22 μm syringe filters	Merck	SLGVV255F
1.5 mL and 2 mL tubes	Eppendorf	30121694
Whatman paper (grade 1)	Merck	WHA1001090
Autoclaved ceramic mortar (300 mL) and pestle	N/A	N/A
Autoclaved glass funnels and metal spatulas	N/A	N/A
Liquid nitrogen	N/A	N/A
*C. reinhardtii* reference sequences	www.github.com/AndreHolzer/IGV-Genomes	v5.6

## Materials and equipment

### Stock solutions

• 1 M Tris, pH 8.0: Dissolve 12.1 *g* of Tris in 60 mL water, and adjust the pH to 8.0 with HCl. Top up to 100 mL with water.
• 1 M HEPES-KOH: Dissolve 38.3 *g* of HEPES in 60 mL water, and adjust the pH to 7.4 with KOH pellets. Top up to 100 mL with water.
• 1 M MgCl_2_: Dissolve 20.33 *g* of MgCl_2_·6H_2_O in water, and top up the solution to a total volume of 100 mL.
• 1 M MOPS: Dissolve 10.46 *g* of MOPS in water, and top up the solution to a total volume of 50 mL.
• 1 M NaCl: Dissolve 5.85 *g* of NaCl in water, and top up the solution to a total volume of 100 mL.
• 1 M KCl: Dissolve 7.45 *g* of KCl in water, and top up the solution to a total volume of 100 mL.
• 500 mM EDTA: Add 18.61 *g* of disodium salt of EDTA to 80 mL of water. Stir the solution on a magnetic stirrer and adjust the pH to 8.0 with NaOH (∼2 *g* of NaOH pellets). Adjust the volume to a total volume of 100 mL.
• 100 mM EGTA: Add 3.804 *g* of EGTA to 80 mL of water. Stir the solution on a magnetic stirrer and adjust the pH to 7.0 with KOH (∼0.5 *g* of KOH pellets). Adjust the volume to 100 mL.
• 2.3 M sucrose solution: Dissolve 78.73 *g* of sucrose in water, and adjust the volume to 100 mL.
• 10% Triton-X 100: Add 45 mL of water to 5 mL of triton-X 100.
• 80% ethanol: Add 10 mL of nuclease free water to 40 mL ethanol.
• 1 X PBS: Add 5 mL of 10 X PBS to 45 mL of water.
• 100 mM Spermidine: Dissolve 1.45 *g* of spermidine in 10 mL of water.
• 1 M DTT: Dissolve 154.2 mg of DTT in water, and adjust the volume to 1 mL with water.

**CRITICAL:** Use nuclease free water to make all stock solutions. Sterilize all stock solutions except 80% ethanol, 100 mM spermidine, and 1 M DTT by autoclaving and store them at room temperature. Sterilize the spermidine and DTT solutions using 0.22 μm syringe filters, and store at ‒20°C. 80% ethanol does not need to be sterilized.

**Table:** Nuclei preparation buffer (NPB)

Reagent	Final concentration	Amount
1 M MOPS	20 mM	2 mL
1 M NaCl	40 mM	4 mL
1 M KCl	90 mM	9 mL
500 mM EDTA	5 mM	1 mL
100 mM EGTA	0.5 mM	0.5 mL
100 mM Spermidine	0.1 mM	0.2 mL
Protease inhibitor tablets	N/A	2
KOH pellets	N/A	Adjust pH to 7.0 to the above contents with KOH
Nuclease free water	N/A	Add to the above contents to make up volume to 100 mL
**Total**	**N/A**	**100 mL**

Make NPB on the day of nuclei preparation and store at 4°C.

**Table:** Nuclei extraction buffer 2 (NEB2)

Reagent	Final concentration	Amount
2.3 M Sucrose	250 mM	2.72 mL
1 M Tris	100 mM	2.5 mL
1 M MgCl_2_	20 mM	0.5 mL
Triton-X 100	1%	2.5 mL
Protease inhibitor tablets	N/A	1
Nuclease free water	N/A	Add to the above contents to make up volume to 25 mL
**Total**	**N/A**	**25 mL**

Make NEB2 on the day of nuclei preparation and store at 4°C.

**Table:** Nuclei extraction buffer 3 (NEB3)

Reagent	Final concentration	Amount
2.3 M Sucrose	2 M	21.7 mL
1 M Tris	100 mM	2.5 mL
1 M MgCl_2_	4 mM	0.1 mL
Triton-X 100	0.15%	0.375 mL
Protease inhibitor tablets	N/A	1
Nuclease free water	N/A	Add to the above contents to make up volume to 25 mL
**Total**	**N/A**	**25 mL**

Make NEB3 on the day of nuclei preparation and store at 4°C.

**Table:** Nuclei storage buffer (NSB)

Reagent	Final concentration	Amount
1 M HEPES	80 mM	2 mL
1 M MgCl_2_	20 mM	0.5 mL
1 M DTT	4 mM	0.1 mL
Glycerol	80%	20 mL
Protease inhibitor tablets	N/A	1
Nuclease free water	N/A	Add to the above contents to make up volume to 25 mL
**Total**	**N/A**	**25 mL**

Make NSB on the day of nuclei preparation and store at 4°C.

**Table:** CTAB buffer

Reagent	Final concentration	Amount
CTAB	55 mM	1 *g*
1 M NaCl	1.4 mM	0.7 mL
500 mM EDTA	20 mM	2 mL
1 M Tris	200 mM	10 mL
β-mercaptoethanol	2%	1 mL
Nuclease free water	N/A	Add to the above contents to make up volume to 50 mL
**Total**	**N/A**	**25 mL**

Make CTAB buffer on the day of DNA extraction and store at room temp.

**Table:** Tagmentation reaction mix

Reagent	Final concentration	Amount
2X Tagmentation buffer^a^	1X	150 μL
Loaded transposase^a^	N/A	13.5 μL
10% tween 20^a^	0.1%	3 μL
2% digitonin	0.01%	1.5 μL
PBS	N/A	99 μL
Nuclease free water	N/A	33 μL
**Total**	**N/A**	**300 μL**

a Use the components provided in the Diagenode ATAC-seq kit.

Make tagmentation reaction mix 5 min before setting up transposase reactions and keep on ice.

**TAP and HS media**: Chlamydomonas culture media is made as described on the Chlamydomonas resource center webpage (http://www.chlamy.org/media.html).

***Note:*** The composition of NPB, NEB2, NEB3 are derived from Bajic et al.,^9^ and NSB recipe is from Sikorskaite et al.^30^

## Step-by-step method details

### Part I: Cell harvesting

**Timing: ∼20 min**

The purpose of this step is to harvest algae cells, concentrate and cryopreserve them in liquid nitrogen to allow for subsequent isolation of their nuclei.

1. Harvest 200 mL of the algal culture (2–3·10^8^ cells) by centrifugation at 3,000 *g* for 5 min at 4°C in 250 mL centrifuge bottles.
2. Resuspend the pellet in 5–10 mL of spent media and transfer the suspension to a 50 mL tube. Spin the suspension at 3,000 *g* for 5 min at 4°C. Discard the supernatant.
3. Flash freeze the pellets in the 50 mL tubes in liquid nitrogen and store at ‒80°C until the day of nuclei isolation.

***Note:*** We recommend continuing with the isolation process directly or shortly after cryopreservation.

### Part II: Cell lysis

**Timing: ∼45 min**

The purpose of this step is to lyse algae cells by manual grinding and the use of chemical detergent to release cellular components.

4. Fill the autoclaved ceramic mortar with 50–75 mL of liquid nitrogen (about ¼ of the mortar).
  ***Note:*** The mortar and pestle do not require to be cooled to 4°C since the contact with liquid nitrogen lowers their temperature instantaneously.
  a. Place a fresh 50 mL tube with 5 mL NPB buffer on ice.
5. Transfer the tube containing the algal pellet from ‒80°C to ice. Dislodge the pellet with a chilled 1 mL pipette tip attached to a pipette and drop into the mortar with liquid nitrogen.

**CRITICAL:** Do not allow the pellet to thaw, and keep the time of transfer of the pellet from ‒80°C to liquid nitrogen minimal (less than 5 min).

6. Pulverize the cell pellet with the pestle in a small amount of liquid nitrogen still present in the mortar (∼10 mL).

***Note:*** Add additional liquid nitrogen to the mortar if pulverization is not complete. Once completed, the pulverized cells appear as a dry green powder. Troubleshooting 1

**Figure 2:**
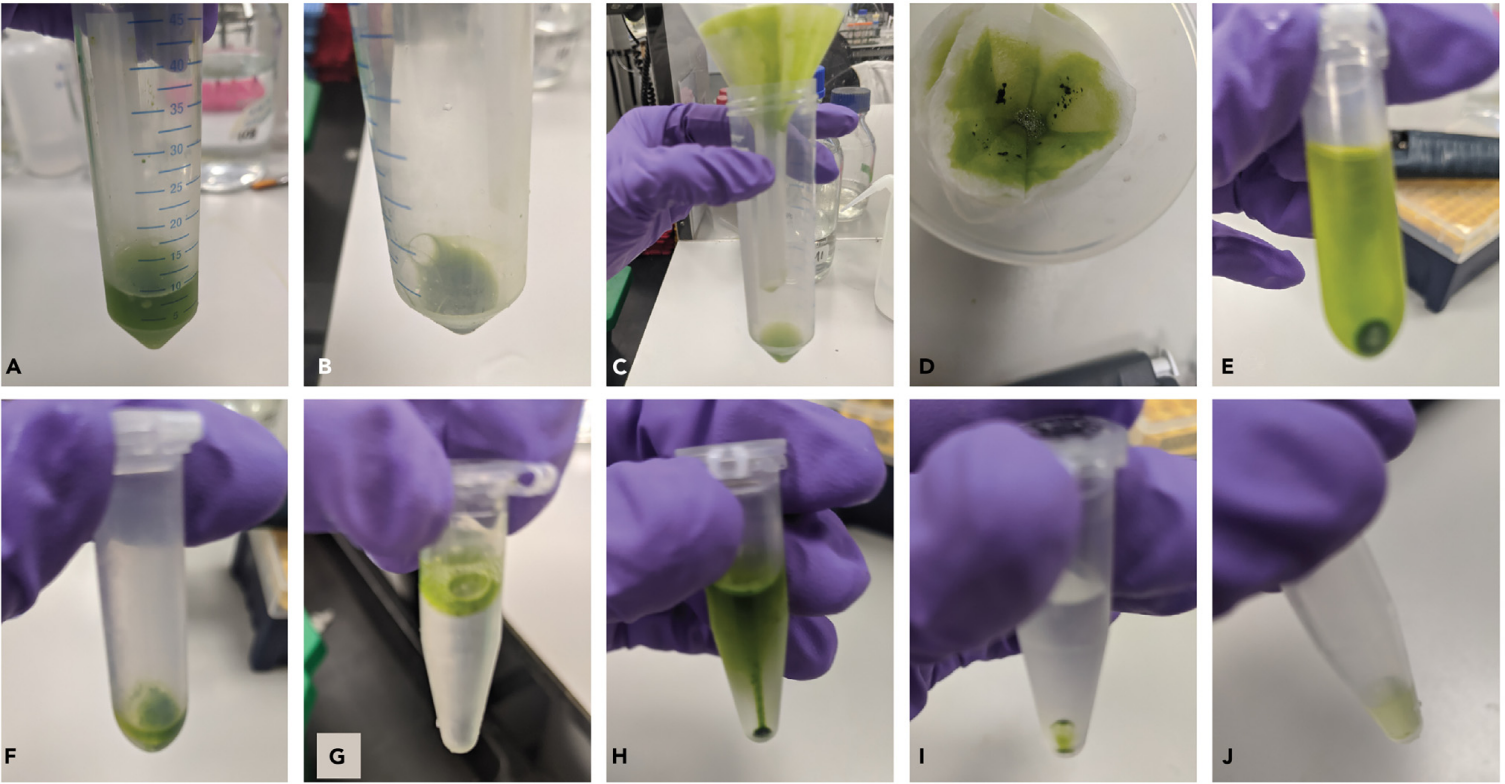
Snapshots from the nuclei isolation protocol (A) Suspension of cells in NPB following pulverization (Step 7) and (B) the resulting pellet after centrifugation steps (Step 8). (C and D) Filtration of the suspension in NEB2 (Step 10). (E) Pellet of cell lysate in NEB2 following centrifugation (Step 11) and (F) after dislodgement (Step 12). (G) Layering of the suspension on NEB3 (Step 12) and (H) the pellet following centrifugation (after Step 12). (I) Nuclei pellet following centrifugation in NPB (Step 17) and (J) the final nuclei suspension in NSB (after Step 18).
8. Spin the suspension in the 50 mL tube at 1,200 *g* for 10 min at 4°C. Discard the supernatant by decanting the tube (Figure 2B).
9. Resuspend the pellet in 1.5 mL of NEB2 buffer in the 50 mL tube using a chilled 1 mL cut-pipette tip.
  a. Place the suspension in 50 mL tube in a 250 mL beaker with ice.
  b. Manually shake the beaker containing the suspension gently (10–15 s shaking alternating with 10–15 s breaks) for a maximum of 3 min.

***Note:*** NEB2 contains 1% Triton X-100, and this is the primary lysis step.

7. Transfer the pulverized cells to the chilled NPB buffer in the 50 mL tube with a chilled spatula, immediately after all the liquid nitrogen vaporized. Add additional 5 mL chilled NPB buffer to the mortar to clear any remaining cells and transfer the contents to the 50 mL tube by decanting (Figure 2A).

### Part III: Separation

**Timing: ∼45 min**

The purpose of this step is to filter and wash the lysate to separate nuclei from intact cells and other contaminants.

10. Overlay a Whatman filter paper cone placed in a chilled funnel with a miracloth cone (Figures 2C and 2D). Filter the above suspension through miracloth and grade 1 Whatman paper.
  ***Note:*** Carry out this filtration at 4°C in a cold room.
  a. Collect the filtrate in a fresh tube. Transfer this filtered suspension to a chilled 2 mL tube.
11. Spin the filtrate from the above step at 12,000 *g*, 4°C for 10 min (Figure 2E). Remove the supernatant using a 1 mL pipette, while being careful not to disturb the pellet.
12. Add 50 μL of NPB to the pellet, and spin at 10,000 *g* for 10 s at 4°C to dislodge the pellet from the tube by placing the tube with the pellet facing the center of the rotor (Figure 2F).
  a. Add 0.3 mL of NEB3 to the dislodged pellet.
  b. Remove the resulting suspension using a 1 mL cut pipette tip and layer atop 0.6 mL of cooled NEB3 in a 1.5 mL tube (Figure 2G).
  c. Spin this tube with layered suspensions at 16,000 *g*, 4°C for 10 min (Figure 2H).
13. Remove the supernatant using a 1 mL pipette.

***Note:*** Since NEB3 is viscous, it is not possible to remove all the supernatant without dislodging the pellet. Allow about 50 μL of supernatant to remain in the tube along with the pellet.

14. Repeat steps 12 and 13.

### Part IV: Clean up

**Timing: ∼30 min**

The purpose of this step is to clean up nuclei using sucrose sedimentation and washing to obtain high quality extracts.

15. Add 20 μL of NPB to the pellet from the last spin and dislodge the pellet by centrifugation as described in step 12.
  a. Add 0.3 mL of 2.3 M sucrose solution to the dislodged pellet.
  b. Layer this suspension on 0.8 mL of chilled 2.3 M sucrose in a 1.5 mL tube.
  c. Spin the tube at 12,000 *g*, 4°C for 10 min.
16. Following the spin, aspirate the supernatant along with the gelatinous green phase and the sucrose cushion layer leaving behind less than 100 μL of liquid.

***Note:*** The pellet is dispersed along the side of the tube, and not condensed at the base of the tube as seen previously with NEB3 spins. The pellet from this step is less green and is not clearly visible.

17. To the tube, add 0.8 mL of NPB, and spin at 12,000 *g*, 4°C for 5 min. Following this wash, the pellet at the base of the tube should become more visible (Figure 2I).
18. Aspirate the supernatant, and resuspend the pellet in 0.1 mL of NSB (Figure 2J). Troubleshooting 2.

### Part V: Quantification of nuclei

**Timing: ∼5 min**

The purpose of this step is to use trypan blue staining and automated counting to allow for a quick quantification of nuclei.

**Figure 3:**
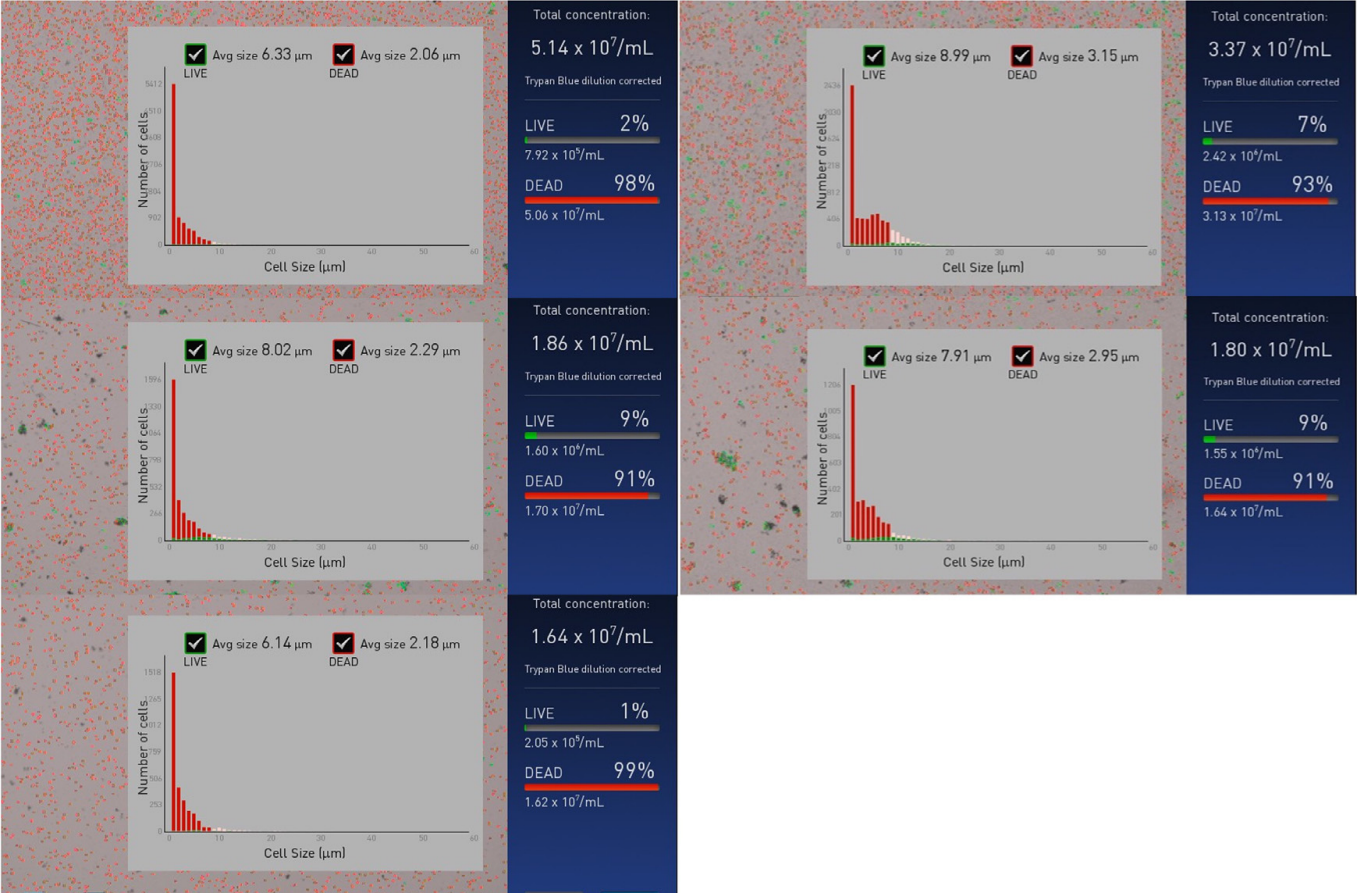
Quantification of nuclei Images captured on the Invitrogen counter of the isolated nuclei (left) from three extracts of cell wall-deficient strain CC-5390 and (right) two samples from walled strain CC-124 of *C. reinhardtii*. Over 90% of the isolates have an average size of 2.0–3.2 μm, indicating that nuclei isolation was successful with little carryover of intact cells. Width of distribution suggests a slightly reduced purity with respect to other organelles for extracts from walled strains.

***Note:*** In parallel, we recommend DAPI staining to investigate the overall level of chloroplast contamination. Following the above described methodology we have found that chloroplast contamination can be slightly more for extracts from cell walled strains. We have demonstrated successful nuclear enrichment and their amenability for use in ATAC-seq is demonstrated by library preparation and ATAC-seq data analysis (Part VI).

***Note:*** Following quantification, we recommend to immediately proceed with the transposase reaction to minimize denaturation or dissociation of DNA-protein complexes.

19. Estimate the concentration and yield of nuclei using the Invitrogen Countess II automated cell counter and Countess cell counting chamber slides.
  a. Dilute 5 μL of the nuclei suspension with 5 μL of trypan blue, and add this 10 μL suspension to a Countess slide. Record the reading on the counter.
  b. The size of the isolates shall predominantly be in the range of 2.0–3.2 μm in diameter which indicates that the isolates are not heavily contaminated by intact cells. Intact cells and chloroplasts are 8–10 μm and 3–4 μm in diameter, respectively. Troubleshooting 3.
  c. In addition to the size average of the isolates, the counter gives a quick estimate of the concentration of nuclei based on the percentage of “dead” cells (Figure 3). Troubleshooting 4.

### Part VI: Downstream ATAC-sequencing

**Timing: >2 days**

While the protocol focuses on the extraction of nuclei from *C. reinhardtii*, the purpose of the steps described below is to highlight how we have used downstream ATAC-sequencing to further assess the quality of the isolated nuclei. The described steps include library preparation, sequencing, and initial data analysis.

20. Resuspend volumes of nuclei corresponding to 55,000 cells (1–4 μL) in 10 μL tagmentation buffer from the Diagenode kit (C01080002) and spin down the solution at 4°C at 10,000 *g* for 5 min. Discard the supernatant, and use the nuclei pellets for setting up transposase reactions.

***Note:*** We also set up a control reaction for denatured DNA using 10 ng of genomic DNA isolated from *C.reinhardtii* cells (2 samples) by CTAB (https://www.chlamycollection.org/methods/minipreps-of-dna-from-chlamydomonas-cultures/)

21. Carry out the transposase reaction, and further steps to make the library for ATAC-sequencing by following the protocol described in the ATAC-seq kit manual from Diagenode (C01080002). Follow the protocol as described in the kit manual.
  a. Set up the transposase reactions at 4°C (on ice) in 1.5 mL tubes by adding 50 μL of tagmentation reaction mix to each of the nuclei and DNA samples.
  b. Suspend nuclei gently in the tagmentation reaction mix by pipetting up and down 2 times.
  c. Transfer the tubes to a thermomixer preheated to 37°C.
  d. Incubate the samples for 30 min at 37°C. Stop the reactions by addition of 0.25 mL of binding buffer provided in the kit. Troubleshooting 5.
22. Purify the transposed DNA following the transposase reaction using columns and reagents provided in the ATAC-seq kit.
  ***Note:*** This purified DNA can be stored at ‒80°C before proceeding to library preparation.
  a. Amplify the purified, transposed DNA with the PCR-mastermix provided in the kit.
  b. Perform qPCR reactions to ascertain the number of additional amplification cycles, and carry out the additional cycles if necessary for each of the samples.
    ***Note:*** Follow the protocols for purification and amplification of transposed DNA and qPCR to ascertain the number of additional amplification cycles as indicated in the ATAC-seq kit manual with the reagents provided in the kit.
23. Purify the amplified libraries using AMPure XP beads, a magnetic rack and 80% ethanol as indicated in the kit manual.
24. Get the libraries sequenced.

***Note:*** Our libraries were sent for low depth sequencing (∼3 million reads per sample) on NovaSeq 6000 at Novogene (UK) Company Limited, Cambridge to obtain 150 bp paired-end reads.

**Figure 4:**
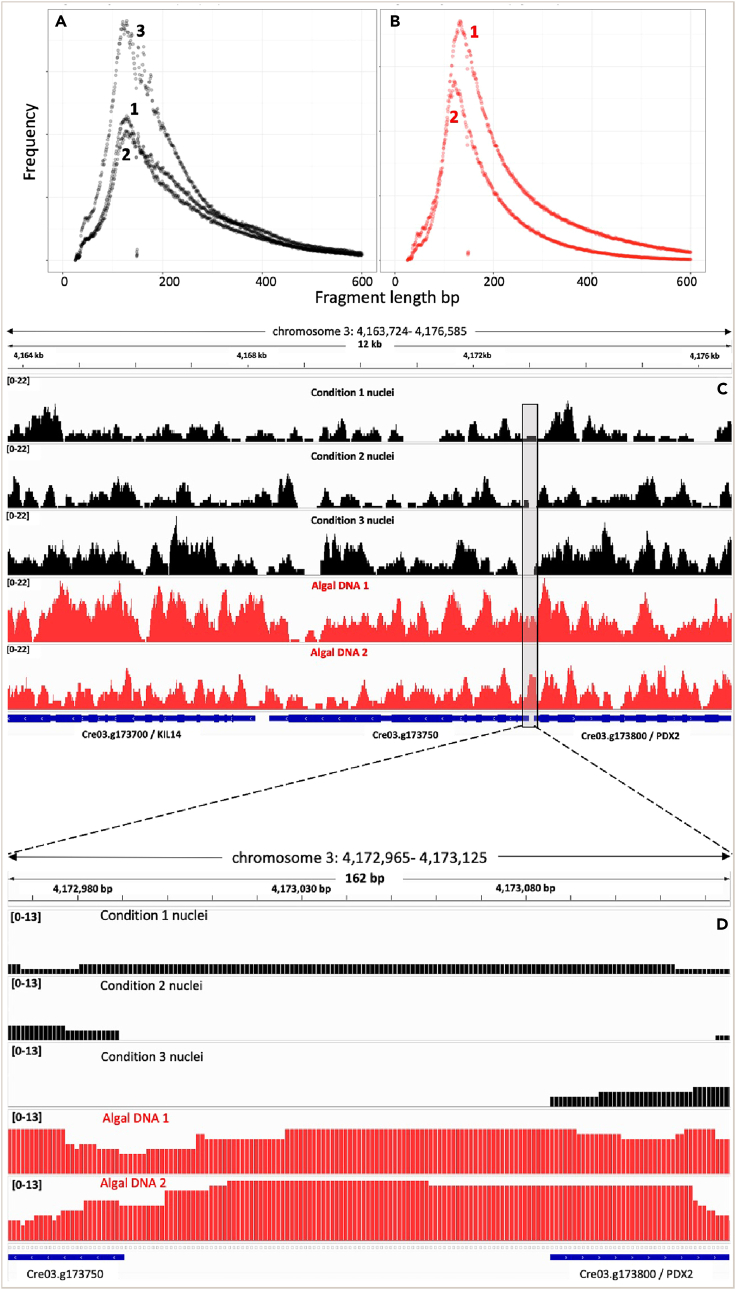
**ATAC-sequencing data** Fragment size distributions calculated following alignment of sequence reads for the ATAC libraries that were made using the three nuclei isolated from CC-5390 in three different conditions (A) and two algal DNA samples(B). All cells for nuclei isolation were grown in 12 h light, 12 h dark cycles. Condition 1 cells were harvested 2 h after onset of light which were grown in the presence of air bubbled through media. Conditions 2 and 3 cells were grown with air and (air+5% CO_2_) respectively, and they were harvested 2 h after onset of darkness. The two algal DNA samples were extracted from CC-5390 cells grown in the presence of constant light with air bubbled through the media. The sequences of ATAC libraries of three nuclei samples and two algal DNA samples were aligned with *C. reinhardtii* genomes using Bowtie2 after removing adaptor sequences using Cutadapt. Representative tracks of the aligned sequences are shown for a region of 12 kb on chromosome 3 of *C. reinhardtii* genome (C). The shaded box region (162 bp) upstream of gene PDX2 in panel C is expanded in D to highlight the differences in accessibility of DNA in the nuclei and DNA samples.

***Note:*** The GC content of *C. reinhardtii* nuclear, chloroplast and mitochondrial DNA are 64.1%, 34.6% and 45.2% respectively.^20^ The difference in GC content between the nuclear and organellar DNA makes it easy to assess the nuclear DNA enrichment from FastQC reports of sequenced reads, even prior to alignment. The GC content of the ATAC-seq reads (FastQC results) and the percentage of reads aligning to nuclear DNA for the samples (determined with SAMtools) obtained in our experiments are listed in Table 1. The data in Table 1 indicate that the nuclear DNA enrichment with respect to organellar DNA was successful.

25. Process the sequenced reads for quality assessment (FastQC),^26^ adaptor removal (Cutadapt with parameters: -e 0.3 -q 20 -m 25),^27^ and alignment to the *C. reinhardtii* reference genome (nuclear genome v5.6, plastid and mitochondrial genome: v4.4) (https://github.com/AndreHolzer/IGV-Genomes) using Bowtie2 (-X 2000 --sensitive --no-mixed --no-discordant -t --fr --seed 120,000,000 --np 2 --mp 7,3).^27^
26. Convert the aligned reads to required formats and analyze using SAMtools,^28^ and visualize alignments using the Integrative Genomics Viewer^29^ (Figures 4C and 4D).

27. Plot the distributions of fragment sizes aligned to the nuclear genome of *C. reinhardtii.*

***Note:*** From our experiments one can see that the graphs for denatured algal DNA and nuclei look different with nuclei graphs showing ‘peaks’ (∼200 bp, ∼400 bp) corresponding to mono- and di-nucleosome fragments. The graphs (Figures 4A and 4B) indicate that the nuclei are intact, and that the isolation method is suitable for ATAC-seq, and other NGS methods such as DNase-seq that require intact nuclei.

**Table 1:** GC content and alignment of ATAC-seq reads

Samples	%GC of reads (paired files)^a^	Percentage of reads aligning to nuclear DNA^b^
1	61%	80%
2	57%	72%
3	62%	94%

a GC content was determined with FastQC.^25^

b Following alignment of reads with Bowtie2,^27^ SAMtools^28^ coverage was used to determine the percentage of reads aligned to nuclear DNA.

## Expected outcomes

Here we briefly describe the key adaptations and findings made with respect to previous protocols.

Both Bajic et al.^9^ and Winck et al.^23^ have used 1% Triton X-100 based buffers for lysis of membranes prior to filtration steps. While Winck et al.^23^ carries out incubation of cells in the lysis buffer for 20 min, Bajic et al.^9^ proceed to centrifugation immediately after resuspension of the pellet in the lysis buffer. We have also used 1% Triton X-100 based buffers while limiting the suspension (Part II: 9) to 3 min in order to allow cell lysis and prevent denaturation of nuclear proteins.

The presented method is different from the previous published protocols in the steps of detergent treatment and filtration (Parts II & III: 7–11). In the two methods that form the basis of this protocol, pulverized cells are filtered prior to treatment with detergent.^9^^,^^23^ Filtration in our protocol is however carried out following detergent treatment. Treatment with detergent following pulverization disrupts organellar membranes releasing fibrous organellar DNA and other intra-organellar contents, most of which would be filtered along with cellular debris, intact cells and large intact chloroplasts. 70 μm nylon^9^ and 22–25 μm^23^ miracloth filters were used previously. Our use of 22–25 μm miracloth and 11 μm grade 1 Whatman filter in combination helps to filter contaminants better.

The resuspension of pellets by pipetting is time consuming and can lead to a loss of material owing to incomplete resuspension, thus we have shortened the duration of this step with a pulse of centrifugation for dislodgement of pellets (Part III & IV: 12–15).

The repetition involving resuspension centrifugation of the isolate in 2 M sucrose containing NEB3 (Part III: 12–13) at 16,000 *g* helps to minimize contamination of nuclei with other cellular components. Bajic et al.^9^ carry out this wash step only once, while Winck et al.^23^ use a buffer of unknown composition (NIBA) for 2 centrifugal washes at 600 *g*.

The use of an automated cell counter for quantification and visualization of nuclei (Part V: 19) is rapid, and helps minimize changes to nucleoprotein complexes before setting up of transposase reactions for ATAC-seq. Fluorescence microscopy and use of hemocytometer as recommended previously^9^^,^^23^ take longer to quantify, and also do not give a population average of the size of isolates. However, they can be helpful to assess the overall quality of extracts.

As result, the isolation of nuclei (2 samples processed simultaneously) from *C. reinhardtii* cell pellets takes around 2 h (with all reagents prepared and cells harvested before processing of samples). Quantification of nuclei takes 3–5 min. The yield from 2‒3·10^8^ algal cells was found to be around 1–5·10^6^ nuclei.

Figure 1 highlights the key adaptations made with respect to previous protocols.

Figure 2 depicts several key steps within the extraction process with 2J displaying a successful extract of nuclei after suspension in the storage buffer.

Figure 3 depicts the expected results after quantification.

Figure 4 depicts the quality of the resulting ATAC-sequencing data.

Table 1 depicts the obtained raw data statistics for ATAC-sequencing of three nuclei extracts.

## Limitations

The protocol has been designed and validated for *C. reinhardtii*, yet it is conceivable that the methodology can be adapted for the extraction of nuclei from various other microalgae. However, we want to acknowledge that adjustments might be necessary to ensure effectiveness for species other than *C. reinhardtii*.

In our methodology we utilize the unique characteristics of *C. reinhardtii*, notably the distinct size difference between its comparatively large chloroplast and its nucleus. This size contrast is instrumental for the rapid assessment of the purity of nuclei isolates using the Countess II cell counter. It is therefore crucial to recognize that the applicability of this metric may be compromised when working with species possessing chloroplasts of sizes comparable to their nuclei. In such instances, we recommend to resort to traditional, albeit slower, microscopy-based techniques to ascertain nuclei purity.

Finally, the lysis of algal cells may not be adaptable for the concurrent processing of more than 2–4 samples. This constraint arises from the requirement for manual grinding using mortar and pestle which poses a practical challenge when aiming to scale up the sample throughput. Thus, we recommend to process samples in batched of 4 or less.

## Troubleshooting

### Problem 1

The pulverized cells in the mortar turn into a green frozen slush which is difficult to transfer to a tube for further processing (related to Part II).

### Potential solution

• This can be avoided by ensuring that the pellet has some remnant liquid nitrogen when the pestle is used to pulverize the cells to a green powder.
• Using a pestle with a wooden handle helps with handling of the cold pestle.

### Problem 2

You want to use a different storage buffer (related to Part IV).

### Potential solution

• Storage buffers for nuclei should be free of any Tris buffer since it undergoes change in pH with temperature. Our storage buffer is therefore free of it. If one chooses to use another storage buffer for nuclei, we recommend choosing one free of Tris.

### Problem 3

Cell counts indicate too many live cells (related to Part V).

### Potential solution

• If working with other algae strains than those tested you might need to extend grinding in liquid nitrogen and/or adjust time of the primary lysis (Part V: 6).
• Also, a second round of filtration through grade 1 Whatman paper (Part V: 7) can be helpful but will likely affect the overall yield.

### Problem 4

Too much contamination can be seen under the microscope (related to Part V).

### Potential solution

• Additional steps of sucrose washing, and subsequent sucrose gradients (Part V: 11–13) can help to further improve the purity of the extracts yet will affect yield and duration of the protocol.

### Problem 5

The sequences of ATAC libraries (after alignment) indicate denaturing of nuclear DNA (related to Part VI).

### Potential solution

• Usage of lower amounts of transposase or reduction in time of transposase reactions can help resolve the issue (Part VI: 21). If the issue remains the isolation shall be repeated.

## Resource availability

### Lead contact

Further information and requests for resources and reagents should be directed to and will be fulfilled by the lead contact, Dr. Andre Holzer (andre.holzer.biotech@gmail.com).

### Technical contact

For technical questions please contact, Dr Indu Santhanagopalan (is438@cam.ac.uk).

### Materials availability

All materials required are stated in the key resources table and materials and equipment section. Composition of the described reagents is also provided.

### Data and code availability

This protocol is focusing on the isolation of nuclei from *C. reinhardtii*. The ATAC-seq datasets/code supporting the downstream applications noted in the protocol have not been deposited in a public repository yet but all data reported in this paper will be shared by the technical contact upon request. In addition, summary statistics describing the raw and processed ATAC-seq datasets are provided as part of this protocol. Any additional information required to reanalyze the data reported in this paper is available from the corresponding authors upon request.
